# The mechanism and therapeutic strategies for neovascular glaucoma secondary to diabetic retinopathy

**DOI:** 10.3389/fendo.2023.1102361

**Published:** 2023-01-23

**Authors:** Yizhen Tang, Yan Shi, Zhigang Fan

**Affiliations:** ^1^ Beijing Ophthalmology and Visual Sciences Key Laboratory, Department of Ophthalmology, Beijing Tongren Hospital, Capital Medical University, Beijing, China; ^2^ Institute of Ophthalmology, Beijing Ophthalmology and Visual Sciences Key Laboratory, Capital Medical University, Beijing, China

**Keywords:** neovascular glaucoma, diabetic retinopathy, pathogenesis, epidemiology, management

## Abstract

Neovascular glaucoma (NVG) is a devastating secondary glaucoma characterized by the appearance of neovascular over the iris and the proliferation of fibrovascular tissue in the anterior chamber angle. Proliferative diabetic retinopathy (PDR) is one of the leading causes of NVG. Currently increasing diabetes population drive the prevalence rate of NVG into a fast-rising lane. The pathogenesis underlying NVG makes it refractory to routine management for other types of glaucoma in clinical practice. The combination of panretinal photocoagulation (PRP), anti-vascular endothelial growth factor (VEGF) injections, anti-glaucoma drugs, surgical intervention as well as blood glucose control is needed. Early diagnosis and aggressive treatment in time are crucial in halting the neovascularization process and preserving vision. This review provides an overview of NVG secondary to diabetic retinopathy (DR), including the epidemiology, pathogenesis and management, so as to provide a better understanding as well as potential therapeutic strategies for future treatment.

## Introduction

Neovascular glaucoma (NVG) is a type of secondary glaucoma that potentially leads to irreversible vision loss and blindness. It was firstly reported by Weiss et al.z in 1963, who observed iris neovascularization in patients with central retinal vein occlusion (CRVO) and proposed the concept of NVG. Thus, NVG is characterized by progressive neovascularization in the iris (NVI) and angle (NVA). Patients usually suffer from sustained severe eye pain, photophobia, high intraocular pressure above 60 mmHg, accompanied by persistent hyperemia, corneal edema, mydriasis, and uveal ectropion. A large number of ocular and systemic disorders could cause NVG, including ischemic conditions, inflammatory conditions, retinal detachment, ocular tumor microenvironment, surgical effect and systemic diseases (1–3). The majority of NVG was secondary to proliferative diabetic retinopathy (PDR), retinal vein occlusion (RVO) and ocular ischemic syndrome (OIS), which causes retinal ischemia/hypoxia and subsequent release of angiogenesis factors. These ischemia and angiogenesis factors drive neovascular growth in the iris and fibrovascular membranes proliferation in the anterior chamber angle, thus blocking the trabecular meshwork, and causing peripheral anterior iris adhesions and progressive closure of the anterior angle. The blockage of aqueous humor drainage eventually leads to a dramatic increase of intraocular pressure, which iteratively aggravates ischemia, destroys anterior chamber function and eventually leads to loss of vision (2). Based on its histological and clinical characteristics, NVG can be divided into three stages: rubeosis iridis, open-angle NVG, and angle-closure NVG. Although NVG can cause severe visual impairment and blindness, it could be controlled and the neovascularization process would be halted in the rubeosis iridis stage if treated promptly and appropriately. Once progressed to the second or third stage, the dysfunction of angle drainage occurs and the management becomes tough.

Proliferative diabetic retinopathy (PDR) is one of the leading causes of NVG, while the underlying pathogenesis of NVG secondary to PDR hasn’t been fully elucidated. Therefore, its management has always been challenging for glaucoma, vitreoretinal and endocrinology specialists in clinical practice. The increasing diabetes population and prevalence of NVG make the situation even more urgent. Consequently, the present article will comprehensively review NVG secondary to diabetic retinopathy (DR) from the aspects of epidemiology, pathogenesis, and management so as to gain a better understanding of the disease and present potential therapeutic targets for future clinical treatment.

## Etiology and epidemiology

Although the prevalence of NVG is relatively low, accounting for 0.7%-5.1% of the overall Asian glaucoma population (4, 5), 5.8% of glaucoma patients in China (6), and about 3.9% of glaucoma patients in Europe (7), it can cause sustained eye pain, devastating glaucomatous optic neuropathy and even blindness (2). It was estimated that the global prevalence of diabetes is about 10% of the total population and diabetes accounts for more than 30% of NVG cases (8). Based on that, proliferative diabetic retinopathy is the leading cause of NVG (9). The prevalence and composition of NVG are different among countries and races (10). In the United States, PDR is the primary cause of NVG, accounting for 52.38% of the population. Other factors are RVO accounting for 36.90%, and unknown factors accounting for 10.71%. In Korea, PDR, OIS, and RVO are the main reasons for NVG, with proportions of 67%, 17%, and 11%, respectively. In China, the reported data demonstrated that 39.7% of NVG was caused by PDR, 22.9% by RVO, and 2.3% by OIS (10).

The clinical feature of NVG due to diabetic retinopathy is also different from the others (11, 12). Patients with CRVO often display tortuous retinal veins, flame-shaped retinal hemorrhage, and a swollen optic disk. OIS patients are generally characterized by dilated but not tortuous retinal veins, dot and blot hemorrhages at the midperipheral retina and absence of hard exudates. While diabetic patients usually display beaded retinal veins, dot and blot hemorrhages at the posterior and midperiphery of the retina, scattered microaneurysms, and retinal exudates. Besides, The retinal arterial perfusion pressure is often decreased in OIS but not in CRVO and PDR.

The association of NVG with diabetic retinopathy is well-acknowledged (13–15). As a secondary systemic disease complication, the disease progression is often slow but irreversible if no early prevention and intervention are made. Studies have confirmed the association between long-term poorly controlled diabetes and the occurrence of NVG (16, 17). Thus NVG is often an advanced manifestation of DR. The reported prevalence of NVG was 2.1% in overall diabetic patients and rose to 21.3% in patients with PDR (13). Besides, NVG is more likely to occur after cataract surgery and vitreoretinal surgery due to surgery-induced inflammation cascade, retinal hypoxia, and the lack of anti-neovascular factors (14, 15). Furthermore, clinical studies have shown that posterior surgery might help the diffusion of vascular endothelial growth factor (VEGF) into the anterior chamber (18). Taking the above risk factors, the incidence of NVG in diabetic patients after ocular surgery raised to 80% (19).

What’s worse, NVG is regarded as a terminal diabetic ocular complication with significant association with diabetic neuropathy/diabetic nephropathy (20, 21). A Logistic regression analysis revealed that HbAlc (p < 0.001) and diabetic nephropathy (p < 0.001) were two significant independent risk factors of NVG (22). Therefore, it’s alert for NVG patients to be aware of poor glucose control and other severe diabetic complications.

## Pathogenesis

In contrast to CRVO, in which the typical NVG occurs within 3 months since the onset of ischemic RVO (so-called ‘100-day glaucoma’),the establishment of hypoxia and ischemia from DR is relatively slow. The major factors causing vascular complications in diabetes are chronic hyperglycemia and ischemia-reperfusion. Studies have found that retinal hypoxia and ischemia lead to the production of a large number of neovascular-related factors (12), resulting in an imbalance between pro-angiogenesis and anti-angiogenesis processes. Normally, angiogenesis factor VEGF and angiopoietin-2 levels are in equilibrium (23). However, under hypoxia and ischemia microenvironment, this balance is broken, shifting to an imbalanced upregulation of VEGF, accompanied by the activation, proliferation, and migration of endothelial cells, pericytes and immune cells. The imbalance thereby stimulates angiogenesis and promotes the formation of neovasculature and neovascular membranes in the fundus, iris, and angle of the anterior chamber, thus blocking and stretching the anterior chamber angle, forcing iris trabecular meshwork adhesion, and eventually causing intraocular pressure elevation and visual impairment. The angiogenesis-related factors involved in the pathogenesis are VEGFs, hepatocyte growth factor (HGF), hypoxia-inducible factor 1-alpha (HIF1a), insulin-like growth factor (IGF), tumor necrosis factor (TNF), inflammatory cytokines (e.g. IL-1β, IL-6, IL-8, etc), pigment epithelium-derived factor (PEDF), transforming growth factor-beta (TGF-β), thrombospondin, and somatostatin, etc (12, 24–26).

**VEGF and angiogenesis.** VEGF is the most widely studied factor implicated in the disease process of NVG (27, 28). It is produced by various cells in the retina (Muller cells, retinal pigment epithelium, pericytes, and ganglion cells) as well as the non-pigmented ciliary epithelium. Importantly, a small amount of VEGF is required in normal eyes to maintain normal ocular blood supply and normal retinal development (29). However, overexpression of VEGF can induce devastating pathological neovascular genesis. Elevated levels of VEGF have been detected in the aqueous humor of patients with NVG secondary to diabetes (30), especially in eyes after ocular surgeries, which might help the diffusion of VEGF into the anterior chamber (18), indicating the critical role of VEGF in the pathogenesis of NVG. Experimental evidence also showed that the injection of human recombinant factor VEGF to primates is sufficient to generate iris neovascularization and NVG (30).

There are mainly five subtypes of VEGF, all of which can bind to specific subtype receptors and stimulate tissue-specific angiogenesis. Among them, VEGF-A is the isoform most closely associated with neovascularization, which inhibits cell apoptosis and capillary degeneration, and participates in the survival of endothelial cells. VEGF-A is markedly increased in the vitreous of PDR patients (31). Hyperglycemia and hypoxia condition activates downstream pathways, thus inducing an inflammation cascade and stimulating the expression of VEGF (32). Cells that produce HIF-1a could also stimulate the release of VEGF-A. Circulating VEGF-A then binds to VEGF receptors on endothelial cells, resulting in the activation of tyrosine kinase pathway and angiogenesis in the tissue (33).

**Hyperglycemia and metabolic alteration.** Studies based on a large population in Singapore and Japan showed a direct association between diabetes and long-term hyperglycemia with increased IOP after the adjustment for central corneal thickness (34, 35), indicating that diabetes might be a risk factor of elevated IOP. Hyperglycemia results in the loss of the pericytes, the apoptosis of the endothelial cells, the thickening of the basement membrane, and cell attachment impairment, which together lead to the breakdown of the blood retina barrier (BRB) (36). These morphological changes in tissue structure greatly strengthen the diffusion of angiogenesis and inflammatory factors, thus triggering subsequent biological processes. Hyperglycemia could also remodel glucose metabolism. The metabolic pathway includes polyol pathway, oxidative stress, protein kinase C (PKC) activation, and advanced glycation endproducts accumulation (37). Glucose is transformed to sorbitol by aldose reductase enzyme *via* the polyol pathway. The accumulation of impermeable sorbital results in pressure changes and osmotic damage to cells. Activation of PKC further accelerates the alteration of basement membrane and vascular permeability. In addition, the formation of advanced glycation endproducts causes the alteration of extracellular matrix proteins, thus exerting accumulated damage on retinal vessels as well as cell death.

**Inflammation and immune response.** Growing evidence suggests that inflammation is a key factor in the pathogenesis of NVG secondary to DR (38, 39), although the detailed molecular mechanism remains ambiguous. Chronic low-grade inflammation is a key driver of capillary occlusion and hypoxia, reinforcing VEGF expression and vascular abnormalities. Several processes, including oxidative stress, ischemia and hyperglycemia contribute to the inflammatory process. Evidence showed that patients with DR have higher levels of inflammatory cytokines (e.g. TNF-α, IL-6, IL-8, and IL-1β) and neurotrophins in their vitreous (40). Moreover, the levels of VEGF-A, IL-8 and EPO in the aqueous humor of NVG patients are significantly higher than that in control groups even received PRP and anti-VEGF therapy (39). Under the inflammatory microenvironment, Muller cells, microglia, astrocytes and T cells become activated, secreting TNF-α, IL-6, IFN-r, MCP-1 and VEGF, inducing endothelial damage and BRB impairment and neurodegeneration (32, 41). Moreover, the level of white blood cell, neutrophil, neutrophil/lymphocyte ratio (NLR), and lymphocyte/monocyte ratio (LMR) were latest found to be associated with NVG process, and NLR is significantly higher in NVG secondary to RVO or DR compared to healthy controls (42), which might present as a potential biomarker for NVG (43).

Studies show that anti-inflammatory drugs such as intravitreal triamcinolone acetonide and NSAIDs reduce VEGF expression and vascular permeability, inhibit retinal cell death, diminish leukostasis, and ultimately improve visual acuity and retinal function (44). Although the pathogenesis of NVG in eyes with uveitis is still unknown, studies indicated that anti-inflammatory treatment can be considered as the first choice for anterior uveitis-associated NVG (45). Targeting microglia for reprogramming of retinal microenvironment could also present a potential therapy for anti-inflammation therapy in the future (46, 47).

## Management of neovascular glaucoma

The management of NVG secondary to DR is a real challenge with a high failure rate (48). NVG usually requires not only medication but also surgery to control the sustained elevated IOP. In adults, bilateral NVG is mostly due to DR (49). For diabetic patients, if NVG occurs in one eye, the other eye is almost inevitable to become NVG without prophylactic pan-retinal photocoagulation (PRP) treatment (49). Therefore, the prompt and intensive management of diabetes is of great importance. A study with long-term observation of 9 years reported that the rates for NVG were 24% in diabetic patients who received conventional treatment, and 8% for those who received intensive treatment (50), indicating that the management does make a difference in the prognosis of the refractory disease.

However, not all eyes with NVG caused by PDR can be directly treated with PRP, and patients with NVG often have significantly lower surgical success rates than other types of glaucoma (51). Previous study reported decreased successful rate of trabeculectomy in NVG secondary to PDR compared to CRVO and OIS (52), which indicated the progressive inflammation in the eyes with PDR as a contributing factor to postoperative scarring and failure. The reported failure rate of medical and surgical intervention of NVG is up to 62.8%, the majority of which suffer from blindness in the end (53). What’s worse, the cost of the treatment is often high. A study in a tertiary hospital in Brazil showed that glaucoma treatment can cost up to 30% of the household income (54). Lower income was associated with worse visual acuity outcomes following NVG surgery (55).

### Management guideline

**Figure 1:**
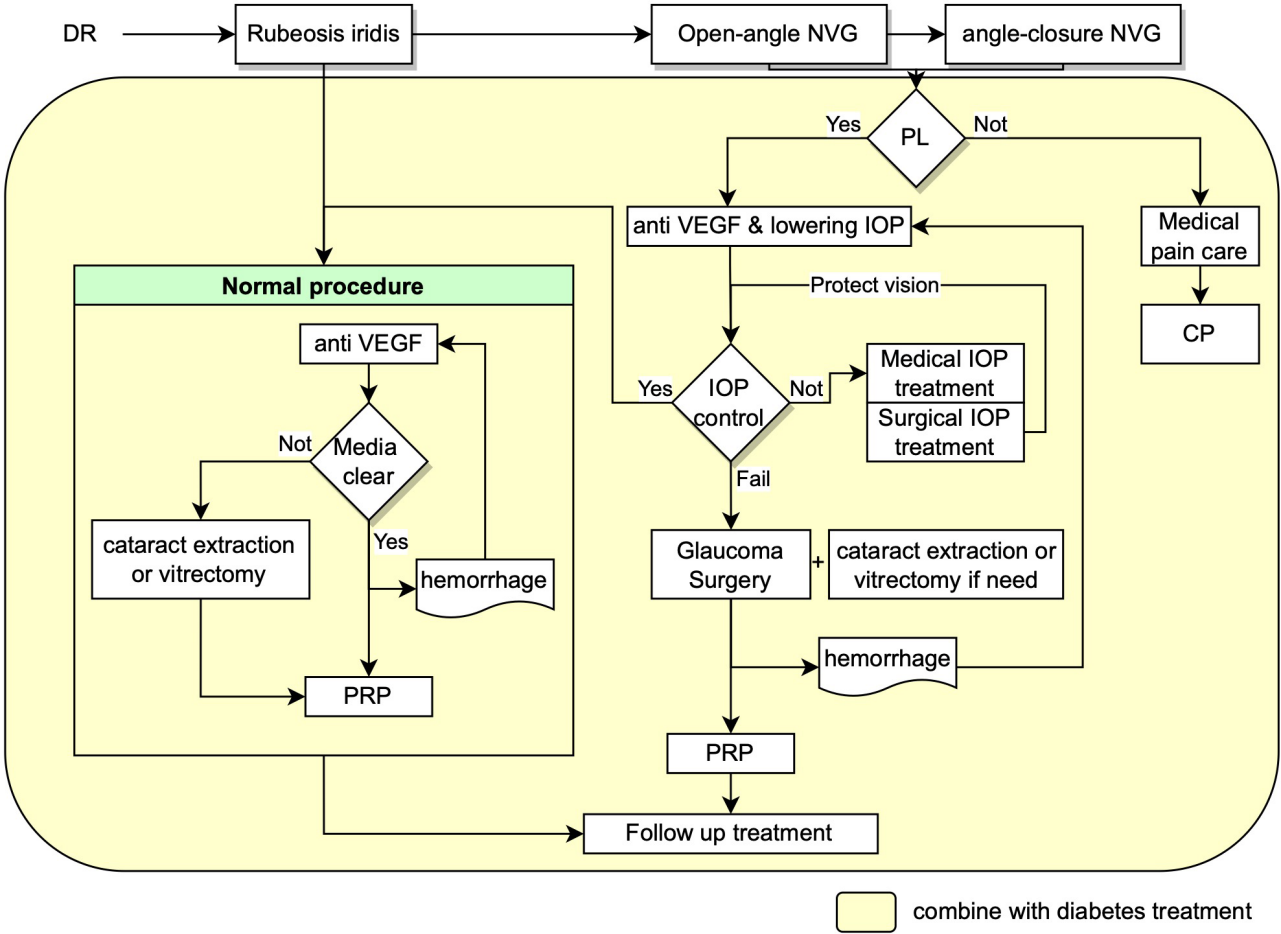
Flow chart showing the recommended management procedures for NVG secondary to DR. NVG: neovascular glaucoma, IOP: intraocular pressure, VEGF: Vascular endothelial growth factor, PRP: panretinal photocoagulation, PL: perception of light, CP: cyclodestructive procedures.

Based on European Glaucoma Society Guidelines and the guideline for NVG in China, early detection of retinal ischemia and treatment of ischemic in time is the most essential and critical management, which minimize the progression of subsequent neovascularization process (56, 57). The treatment and management of NVG secondary to PDR require careful and systematic work, with a team of glaucoma, vitreoretinal and endocrinology specialists to control the blood glucose, IOP and retinal ischemia condition etc at the same time. Management of NVG focus on mainly two aspects as shown in **Figure 1**: treatment of neovascularization and intraocular pressure. The final goal is to maximize the preservation of visual function with approaches including panretinal photocoagulation (PRP), anti-vascular endothelial growth factor (VEGF) therapy, anti-glaucoma therapy including drug therapy and surgical interventions, management of the systemic disease and intensive follow-up at the same time (12, 51, 58, 59).

### Treatment of neovascularization

The treatment of retinal ischemia consists of pan-retinal photocoagulation (PRP) and intravitreal anti-VEGF injections (27). Drugs such as aflibercept, bevacizumab, ranibizumab, pegaptanib, and brolucizumab could suppress the expression of VEGF and therefore hinder the neovascularization process.

**Anti-VEGF treatment.** A case report showed that intravitreal aflibercept (2 mg into the vitreous body on the first day, 4 weeks, 8 weeks, and then every 8 weeks until 52 weeks) may be an effective treatment for the first and second stage of NVG, presenting rapid and sustained regression of NVI and NVA and well-controlled IOP (60). Periodic anti-VEGF treatment leads to more rapid regression of neovascularization than PRP and might be an appropriate therapy prior to any surgical treatment of NVG. However, each dose of anti-VEGF injection could only last for up to six weeks and the penetration distance limits its efficiency in working on the neovascular in the iridocorneal angle. Researchers are working on this problem by exploring novel agents. For instance, brolucizumab has the lightest molar mass (26 kD). Smaller molar mass enables it with higher delivery concentration to work on retinal tissue. Nevertheless, further studies are needed to optimize the delivery method, dose, timing, and agent for anti-VEGF administration.

The intravitreal injections of anti-VEGF should be administrated prior to PRP and/or surgical IOP control since the suppression iris and angle neovascularization only lasts for approximately 3-6 weeks with anti-VEGF injections, which preserves the time for adequate PRP and/or glaucoma surgery to be conducted (61). Furthermore, Somatostatin would inhibit the signal transduction pathway of IGF-1 which is upstream of VEGF, thus resulting in decreased VEGF production (62).

**Pan-retinal photocoagulation (PRP).** PRP is a well-acknowledged procedure for ischemic retinal conditions, and it is believed to reduce anterior segment neovascularization and prevent the development of NVG in diabetic retinopathy. It’s recommended to create every possible condition to complete PRP as soon as possible. If PRP cannot be directly performed due to the opacity of the refractive medium, intravitreal anti-VEGF injections and surgeries to restore the transparency of the refractive medium should be performed (63) to create conditions for PRP, including cataract extraction or vitrectomy combined intraocular PRP. If treated promptly at the early stage, it’s possible that the neovascular would regress and the neovascularization process would be halted. One study demonstrated that intravitreal triamcinolone prior to PRP improved the effect of PRP in eyes with PDR by alleviating NV and retinal thickening (64). Besides, topical steroids and cycloplegics can be used for PRP to control inflammation and improve comfort.

### Treatment of high intraocular pressure

Neovascular glaucoma requires aggressive intervention to lower intraocular pressure (65). Every possible measure should be taken to reduce intraocular pressure, including anterior chamber puncture, systemic or topical application of ocular hypotensive drugs and anti-glaucoma surgery (57, 58).

#### Medical treatment

The medications for NVG mainly include IOP-lowering drug such as carbonic anhydrase inhibitors, beta-blockers, and alpha-2 agonists. Prostaglandin agents are not recommended since they accelerate inflammation. Miotics should also be avoided because they may increase the permeability of the blood-aqueous barrier capillaries therefore aggravating inflammatory response. Moreover, topical steroids and cycloplegics can be used to alleviate inflammation and improve patients’ comfort. Other medications such as hyperosmotic agents (mannitol) can be administered temporally to reduce IOP (49, 66, 67).

#### Surgical treatment

In most cases of angle-closure NVG, medical therapy would be insufficient to control IOP and prevent further visual loss. Once the dysfunction of angle drainage happens, neovascular glaucoma is refractory to medication intervention alone. The iridocorneal angle is altered by neovascularization. Surgery therapy for NVG includes trabeculectomy combined with antimetabolite, glaucoma drainage devices, cyclophotocoagulation and cyclocryotherapy.

**Trabeculectomy**, also known as glaucoma filtration surgery, is less efficient for NVG due to the severe inflammation of NVG, scar formation and unavoidable post-surgery complications. Importantly, VEGF does not only participate in angiogenesis but also involves in the process of wound healing and epithelialization. In addition, there are some evidence showing the high concentration of VEGF in the tenon tissue of patients with failed surgery, which may also account for the high failure rate of trabeculectomy for NVG (12).

**Glaucoma drainage devices** include valved and non-valved implants. Valved implants are recommended for NVG because of their high efficiency and safety in reducing IOP. Ahmed glaucoma valve (AGV), which was created by Mateen Ahmed and approved by FDA US in 1993, has a better mechanism to control IOP and is widely used in clinical practice. Ahmed valve consists of a plate, a drainage tube and a valve. Currently, there are at least eleven available models of Ahmed valves depending on single and double plate, pars plana or pars plana pediatric, and others (59). Numerous studies support that AGV implantation is efficient for refractory glaucoma like NVG (59). Some may worry about its postoperative complications such as cornea edema, damage of the corneal endothelial cells, exposure of the drainage tube, fibrosis around the plate, etc. However, with appropriate surgery procedures, these complications could be reduced to the minimum. We previously proposed modified procedures for AGV implantation and achieved decent clinical outcomes (68). The key point is the effective utilization of the posterior episcleral space and the minimum disturbance of the fascia around the drainage valve disc, thus avoiding the formation of fibrosis. Generally, a conjunctival incision was selected at 8mm behind the limbus and the disc was fixed at 10 mm behind limbus in the upper temporal region of the eyeball. The scleral flap and scleral tunnel are designed to ensure that at least 8 mm drainage tube is fully buried under scleral layers, which effectively reduces the possibility of drainage tube exposure and tube moving during eyeball movement, then reduce the incidence of corneal endothelial decompensation. Moreover, covering the drainage tube with an autologous scleral flap avoids possible rejection response, therefore results in fast postoperative recovery. In addition, the end of the drainage tube is cut into a bevel, which is convenient for the drainage tube to enter the eye through the channel. More importantly, it prevents the drainage tube from contacting the corneal endothelium and prohibits it from being blocked. Theoretically, a successful AGV implantation could keep a stable postoperative intraocular pressure below 12 mmHg. A meta-analysis comparing the efficacy of management for NVG has shown that Ahmed valves achieved better visual acuity as compared to the other devices (69), indicating AGV as an efficient surgical method of NVG. Similar to trabeculectomy, a higher concentration of VEGF in the tenon tissue may also account for the high failure rate of AGV implantation for NVG (12). While we should also be alert that the wound healing process would be slow in patients with diabetes after successful anti-VEGF treatment, especially in older people, which causes wound leakage and bleb-related complications.

**Minimally invasive glaucoma surgery (MIGS)**. Recently, increasing attention has been drawn to MIGS, a revolution in glaucoma surgery with minimal incision and a faster recovery time. There are various categories of MIGS, including the aqueous shunt, Ex-PRESS shunt, XEN gel stent, etc. But their efficiency on NVG needs further validation.

**Cyclodestructive procedures**. Cyclodestructive procedures are the last resort of NVG patient resistant to medical and surgical treatment, which include cyclocryocoagulation, cyclodiathermy, and trans-scleral cyclophotocoagulation. These procedures would damage the ciliary epithelium and stroma by reducing aqueous humor production. It might also cause serious complications like inflammation and atrophia bulbi (70, 71). However, cyclophotocoagulation (CPC) is still another widely applied option for clinicians, which has been proved as an effective treatment for lowering IOP and relieving pain in advanced cases of NVG (72, 73). Recently, the micropulse transscleral cyclophotocoagulation (MP-TSCPC) has been developed declaring less damage to the ciliary body (74). Increasing studies support MP-TSCPC as a successful technique to reduce IOP in refractory glaucoma with substantially less severe complications compared to traditional cyclodestructive procedures (75–77).

## Conclusion

In summary, intensive and aggressive monitoring of blood glucose and the primary disease should be of the highest priority for patients with NVG secondary to DR. Besides, the combination of intraocular anti-VEGF injection, PRP in time, and prompt IOP control offer routine management to halt NVG progression and preserve vision. Furthermore, unveiling the underlying pathology of NVG secondary to DR is of great significance to potential medical interventions. Novel cytokines towards anti-neovascularization and anti-inflammation processes need further investigation and validation.

## Author contributions

YT did the literature review and drafted the manuscript. YS drafted and revised the manuscript. ZF designed and revised the manuscript, and provided financial support for the paper. All authors contributed to the article and approved the submitted version.
